# Towards the Use of Natural Compounds for Crop Protection and Food Safety

**DOI:** 10.3390/foods11050648

**Published:** 2022-02-23

**Authors:** Lisa I. Pilkington

**Affiliations:** School of Chemical Sciences, University of Auckland, Auckland 1010, New Zealand; lisa.pilkington@auckland.ac.nz; Tel.: +64-9-373-7599 (ext. 86776)

The six research articles/communications and one review that comprise this Special Issue, which concerns studies towards natural compound use for crop protection and food safety purposes, highlight the most recent research and investigations into this exciting area.

With an ever-increasing global population, the demand for food, and on food-production, is massive. It is critical that we are able to meet this demand and mitigate the risks and factors that challenge our ability to do so, including pestilence to food crops and biological threats to food safety, before food reaches the consumer. As such, the advancement of measures to both protect crops and facilitate the surety of safe food products to end-users is a research area of great interest and growing development. 

Essential oils are concentrated volatile extracts from plants and are, thus, a mixture of natural products. The application and use of EOs is seen as a method to increase the shelf-life of highly perishable foods by inhibiting pathogen proliferation. Due to the increasing interest in their use, Angane and co-workers [1] review recent research on EOs, focusing on the antibacterial activity of fruit-peel EOs, and the mechanism of action of EO components, as well as providing an overview of the recent contributions of EOs in food matrices. As stated in the review, research to date is extremely encouraging, and further research to devise strategies for EO application at an industrial scale is recommended.

As a specific example of essential oils and recent research into them, Raveau et al. [2] assess clary sage and coriander essential oils for their antifungal, herbicidal and insecticidal activities against notable plant pathogens and pests. It was found that these essential oils were able to inhibit the growth of a range of fungi, and that they exerted anti-germinative, herbicidal, repellent and fumigant effects. Furthermore, it was found that essential oil made from the aerial section of coriander exhibited the most significant antifungal and herbicidal effects. This work highlights that these essential oils have notable potential to act as crop protectants and control post-harvest decay. 

Linalool is a vital component of many essential oils and has been particularly noted for its activity against *Listeria monocytogenes* (LM), one of the most serious foodborne pathogens that is responsible for the onset of listeriosis. In their work, Gao et al. [3] conduct an iTRAQ-based quantitative proteomic analysis to investigate the response of LM when exposed to linalool, in order to ascertain information about linalool’s mode of action. GO and Kyoto Encyclopedia of Genes (KEGG) enrichment analysis, in conjunction with flow cytometry data, presented cell membranes, cell walls, nucleoids and ribosomes as putative targets of linalool against LM. 

In addition to essential oils and their components, other natural chemicals and procedures are also being explored to prolong the shelf-life and traits of produced foods. An example of this includes the study of the use and effects of carbon dioxide treatment to control warehouse pests that commonly affect dried apricots, by Sadeghi et al. [4] In their research article, this group describes their investigation into the use of carbon dioxide gas at varying pressures to control two pest species: *Tribolium castaneum* (Herbst) and *Rhyzopertha dominica* (F.). In addition, the effect of CO_2_ gas on the quality characteristics of dried apricots were assessed. Overall, it was found that CO_2_ gas has the potential to protect dried apricots from *T. castaneum* and *R. dominica* during storage, without any significant impact on the product quality.

The storage and quality, this time of meagre fillets after being treated by cold-smoking, were explored by Messina and co-workers [5]. The smoked fillets were stored for 35 days at 4 °C, after which a range of analyses were performed to assess their sensory, biochemical, physical–chemical and microbiological properties. The results showed positive effects on the fillets’ biochemical parameters and lipid peroxidation. Overall, this work highlights the potential to produce cold-smoked meagre as a value-added fish product. 

In order to meet the rising demand for products, a range of methods are being explored to increase crop yield and production. Biostimulation is one such technique, and Rouphael et al. [6] explore the effect of plant biostimulation on fruits of the traditional tomato germplasm, which has been largely unexplored until now. They investigated how a tropical plant-derived biostimulant influenced the nutritional, functional, and compositional characteristics of tomato fruits, by profiling primary and secondary metabolites in the fruit. Biostimulation affected fruits from the different landraces differently, in many cases leading to improved yield and fruit quality, thus highlighting biostimulation as a promising method to optimise fruit yield and quality.

Jiménez-Gómez et al. [7] explore another potential method to increase crop production: the replacement of chemical fertilisers with biofertilisers (including plant-root-associated beneficial bacteria). In their research article, they describe their work, which assesses the use of *B. halotolerans* SCCPVE07 and *R. laguerreae* PEPV40 strains as efficient biofertilisers for escarole crops. It was shown that these two strains promoted plant development, and the escarole plants showed an increase in a range of minerals and constituents.

The innovative and exciting research included in this Special Issue highlights the interest and potential of this emerging area, addressing some of the most pressing global issues that we face, including sustainably feeding our ever-increasing population.
